# A Meta-Analysis of the Clinical Use of Curcumin for Irritable Bowel Syndrome (IBS)

**DOI:** 10.3390/jcm7100298

**Published:** 2018-09-22

**Authors:** Qin Xiang Ng, Alex Yu Sen Soh, Wayren Loke, Nandini Venkatanarayanan, Donovan Yutong Lim, Wee-Song Yeo

**Affiliations:** 1 National University Hospital, National University Health System, Singapore 119074, Singapore; alex_ys_soh@nuhs.edu.sg (A.Y.S.S.); paeyws@nus.edu.sg (W.-S.Y.); 2 MOH Holdings Pte Ltd., 1 Maritime Square, Singapore 099253, Singapore; wayren.loke@mohh.com.sg; 3 University of Nottingham Medical School, Queen’s Medical Centre, Nottingham NG7 2UH, UK; nandini.venkatanarayanan@nuh.nhs.uk; 4 Institute of Mental Health, Buangkok Green Medical Park, 10 Buangkok View, Singapore 539747, Singapore; Donovan_LIM@imh.com.sg

**Keywords:** IBS, irritable bowel syndrome, functional, curcumin, turmeric, Indian saffron, natural product

## Abstract

Irritable bowel syndrome (IBS) remains a prevalent and difficult-to-manage gastrointestinal condition. There is growing interest in the use of traditional medicine to manage IBS. In particular, curcumin, a biologically active phytochemical, has demonstrated anti-inflammatory and anti-oxidant properties and mucosal protective effects in rat models of colitis. This meta-analysis thus aimed to investigate the hypothesis that curcumin improves IBS symptoms. Using the keywords (curcumin OR turmeric OR Indian saffron OR diferuloylmethane OR curcuminoid) AND (irritable bowel syndrome OR IBS), a preliminary search on the PubMed, Medline, Embase, PsychINFO, Web of Science, and Google Scholar databases yielded 1080 papers published in English between 1 January 1988 and 1 May 2018. Five randomized, controlled trials were systematically reviewed and 3 were included in the final meta-analysis. Random-effects meta-analysis based on three studies and 326 patients found curcumin to have a beneficial albeit not statistically significant effect on IBS symptoms (pooled standardized mean difference from baseline IBS severity rating −0.466, 95% CI: −1.113 to 0.182, *p* = 0.158). This is the first meta-analysis to examine the use of curcumin in IBS. With its unique anti-oxidant and anti-inflammatory activities and ability to modulate gut microbiota, curcumin is a potentially useful addition to our armamentarium of agents for IBS. It also appears safe and well-tolerated, with no adverse events reported in the available trials. However, current findings are based on a considerably limited evidence base with marked heterogeneity. More robust clinical trials involving a standardized curcumin preparation and larger sample sizes should be encouraged.

## 1. Introduction

Irritable bowel syndrome (IBS) is an exceedingly common and debilitating gastrointestinal condition, characterized by chronic abdominal pain and a change in the frequency or form of stool [1]. It affects an estimated 10% to 15% of the global population [2] and carries a significant disease burden in terms of decreased productivity, increased healthcare costs and reduced health-related quality of life [3]. 

Despite the prevalence of IBS, its pathophysiology remains incompletely understood. Increasing research on the human microbiome has highlighted the role of gut microbial dysbiosis in IBS [4]. Studies on post-infectious IBS have provided etiological insights into the pathogenesis of IBS. It is well documented that following infective gastroenteritis, more than 10% of affected individuals go on to develop post-infectious IBS [5]. A consequence of infective gastroenteritis is the disruption of normal gut flora. Though the gut microbiota is known to have inter-individual variations, relative increase in *Bacteroides* and *Prevotella* bacteria have been frequently reported in patients with IBS compared to healthy controls [6]. The microbiome is thought to modulate inflammation and act either directly or indirectly through microbial metabolites. Probiotics [7], rifaximin [8], and other attempts to restore normal gut flora have been demonstrated to alleviate IBS symptoms [9]. However, repeated courses of treatment are often required, raising cost concerns. Current therapies are also limited and based on a weak evidence base [10].

Research efforts to find new and more effective gut microbiota-based therapies are ongoing. There is growing enthusiasm in the use of traditional medicine and many patients with IBS frequently turn to complementary and alternative (CAM) therapies [11]. Curcumin (diferuloylmethane), a bright yellow phytochemical, is the main curcuminoid found in turmeric (*Curcuma longa*), a popular spice used during food preparation in South Asia and the Middle East [12]. Curcumin has long been used in Ayurvedic medicine to treat various inflammatory conditions e.g., arthritis and ulcers [13]. It is also found in the traditional Chinese medicine Jieyu-wan and Xiaoyao-san, which are prescribed to manage stress and mood disorders [14].

Modern studies on the pharmacology and use of curcumin have reported its potent anti-oxidant [15], anti-inflammatory [16] and anti-depressant [17] effects. It also has demonstrated mucosal protective effects in rat models of colitis [18] and reported efficacy in patients with inflammatory bowel disease [19]. Curcumin also has poor bioavailability [20] and majority of it is excreted in the faeces unmetabolized [21]. This means that after ingestion, curcumin reaches the gut almost unaltered and could exert potentially beneficial effects on the gut microbiota. As no meta-analysis has been done up till now to investigate the efficacy of curcumin in patients with IBS, this meta-analysis is, thus, timely and necessary to summarise current evidence and generate hypotheses for further research.

## 2. Methods

A systematic literature search was conducted in accordance with the Preferred Reporting Items for Systematic Reviews and Meta-Analyses (PRISMA) guidelines. Using the keywords (curcumin OR turmeric OR Indian saffron OR diferuloylmethane OR curcuminoid) AND (irritable bowel syndrome OR IBS), a preliminary search on the PubMed, Medline, Embase, PsychINFO, Web of Science and Google Scholar databases yielded 1080 papers published in English between 1 January 1988 and 1 May 2018. Grey literature was searched using Google search. Title/abstract screening were performed independently by three researchers (Q.X.N., W.Y.L., and N.V.) to identify articles of interest. For relevant abstracts, full articles were obtained, reviewed and also checked for references of interest. If necessary, the authors of the articles were contacted to provide additional data. 

Full articles were obtained for all selected abstracts and reviewed by three researchers (Q.X.N., W.Y.L., and N.V) for inclusion. Any disagreement was resolved by discussion and consensus. The inclusion criteria for this review were: (1) published randomized, controlled trial, (2) curcumin administered as an active intervention, (3) study participants with IBS, and (4) available outcome measures for treatment efficacy. Trials that were not placebo-controlled were excluded as a high placebo response rate is known for clinical trials involving patients with IBS [22].

Methodological quality of the eligible clinical trials was appraised using the Cochrane Collaboration’s tool for assessing risk of bias [23]. As different scales and scoring systems, e.g., the IBS symptom-related quality of life (IBSQOL) and IBS Symptom Severity Score (IBS-SSS), were used in the various studies to assess IBS severity pre- and post-intervention, the primary outcome measure of interest was the standardized mean difference (SMD) for mean reduction in IBS symptoms from baseline with curcumin, per-protocol analysis. Estimates were pooled and where appropriate, 95% confidence intervals (95% CI) and *p*-values were calculated. 

Heterogeneity amongst the different studies pooled was examined using the *I*^2^ statistic and Cochran’s *Q* test. *I*^2^ > 50% indicates substantial heterogeneity. Due to the small number of studies available, a funnel plot and sensitivity analyses were not done. All analyses were done using MedCalc Statistical Software version 14.8.1 (MedCalc Software bvba, Ostend, Belgium; http://www.medcalc.org; 2014). 

## 3. Results

**Figure 1:**
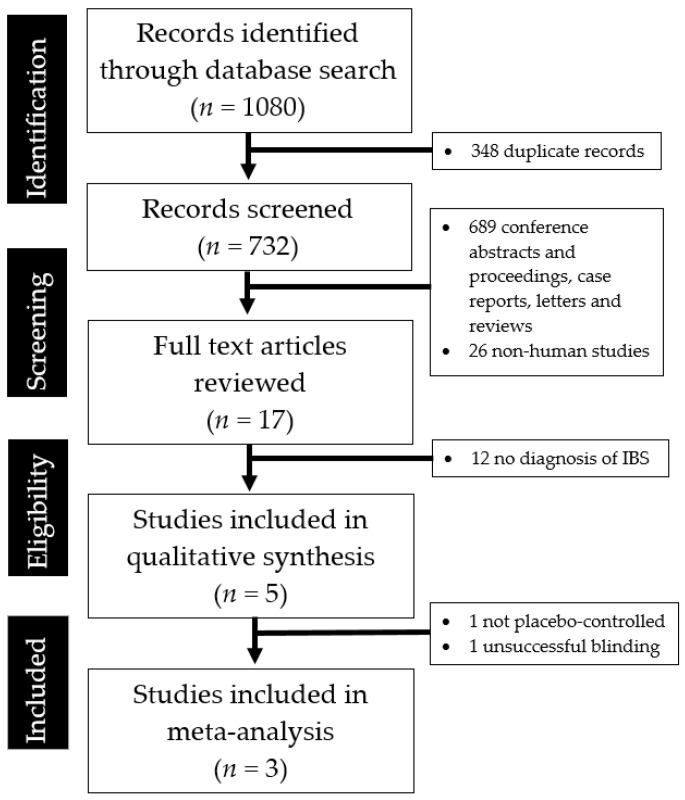
PRISMA flow diagram showing the studies identified during the literature search and abstraction process.

Of the 1080 citations retrieved, 17 full papers were selected for further review. Five studies were systematically reviewed and their key findings were summarised in Table 1. A total of three studies with a total of 326 individuals with IBS were included in the final meta-analysis. One study [24] was excluded as it was only partially blinded and was not placebo-controlled, while another study [25] was excluded as the sample size was limited and there was a high drop-out rate (11 out of 32 participants) and consequent loss of statistical power. Patient blinding was also unsuccessful in that study, as the majority of the patients correctly identified the active intervention or placebo during the trial. Further reason for exclusion from the meta-analysis is that the study [25] investigated a curcumin-containing Ayurvedic preparation with a proportionately small amount of turmeric compared to the other constituents (curry (*Murraya koenigii*), pomegranate (*Punica granatum*), and turmeric (*Curcuma longa* rhizome pulvis) in a 6:3:1 ratio). The abstraction process and reasons for exclusion were detailed in Figure 1. None of the authors had to be contacted to provide additional data.

Methodological quality of the eligible clinical trials was appraised using the Cochrane Collaboration’s tool for assessing risk of bias as shown in Table 2.

**Figure 2:**
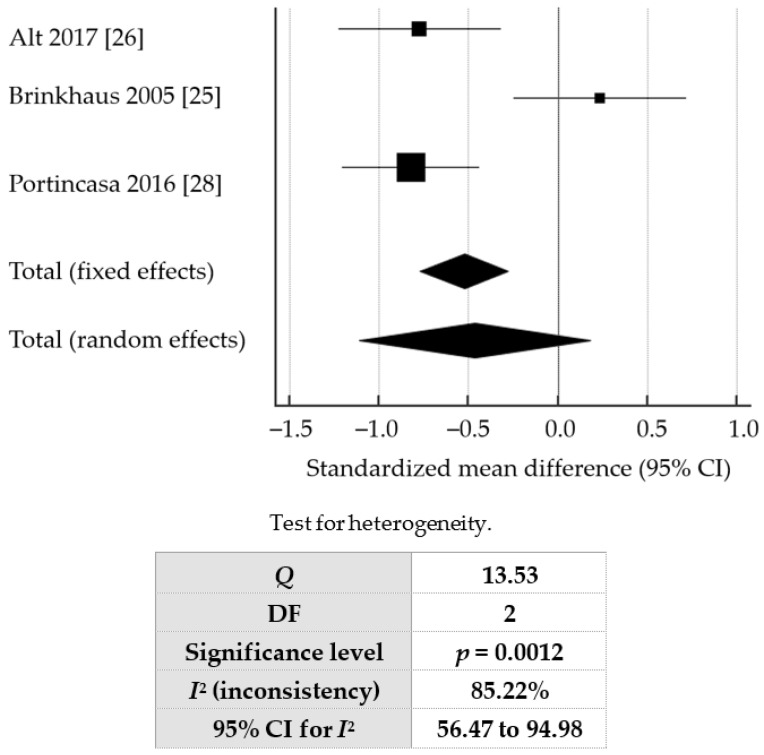
Forest plot showing overall standardized mean difference (SMD) for change in baseline IBS severity rating.

Applying per-protocol analysis and a random-effects model, the pooled SMD from baseline IBS severity rating was −0.466 (95% CI: −1.113 to 0.182, *p* = 0.158), which showed a beneficial, albeit not statistically significant, effect of curcumin on IBS symptoms, compared to placebo. The forest plot is shown in Figure 2. A random-effects model was applied due to the high degree of heterogeneity observed (*I*^2^ = 85.22%).

## 4. Discussion

Overall, random-effects meta-analysis based on three studies and 326 subjects found curcumin to have beneficial but not statistically significant effects on IBS symptoms. Of the five clinical trials reviewed, three reported positive and significant effects for curcumin-containing products [24,26,28], while two [25,27] found no significant effects compared to placebo and advised against monotherapy with curcumin. It is important to note that in the study by Brinkhaus et al. [25], diagnosis of IBS was not based on the established Rome Criteria [29], but rather an intensive clinical examination ruling out other organic causes. The study also recruited particularly treatment-refractory patients with IBS; most of them had a diagnosis of IBS for an average of seven years prior to the study, have had abdominal complaints for an average of 15 years and previous treatments were unsuccessful. This could have accounted for the apparent lack of effect observed for curcumin in the abovementioned study [25]. In the other study by Lauche et al. [27], which found non-significant effects compared to placebo, the sample size was limited and there was a high drop-out rate (11 out of 32 participants) and consequent loss of statistical power. Patient blinding was also unsuccessful in that study [27], as the majority of the patients correctly identified the active intervention or placebo during the trial. It was encouraging that curcumin had demonstrated positive effects in the other two placebo-controlled trials [24,28], which recruited male and female patients with at least moderate symptom severity (IBS-SSS ranging from 250 to 300).

To the best of our knowledge, no prior meta-analysis has been done to investigate the efficacy of curcumin on IBS symptoms. Although the meta-analysis did not find an overall statistically significant effect, the potentially relevant properties of curcumin for IBS sufferers may still have clinical significance or utility. Curcumin’s beneficial effects in IBS are likely related to its unique combination of anti-oxidant [15] and anti-inflammatory [16] activities. Curcumin has been shown to attenuate circulating interleukin-6 (IL-6) levels [30] and regulate key mediators of cellular inflammation, including 5-lipoxygenase (5-LOX), cyclooxygenase-2 (COX-2) and inducible nitric oxide synthase (iNOS) [31]. Current studies on patients with IBS have highlighted a pro-inflammatory phenotype in these patients, with higher circulating IL-6 levels, immune activation and chronic, low-grade, subclinical mucosal inflammation [32]. Curcumin also has demonstrated mucosal protective effects in rat models of colitis [18] and reported efficacy, even in patients with inflammatory bowel disease [19]. 

A recent study using a rat model of IBS also found that curcumin was able to exert beneficial effects via the “brain-gut” axis [33]. Curcumin administration increased serotonin (5-HT), brain-derived neurotrophic factor (BDNF) and phosphorylation of cAMP response element-binding protein (pCREB) expression in the hippocampus and colon [33]. Psychosocial factors have been implicated in the etiology of IBS [34]. Stress is thought to potentiate immune activation as it stimulates pro-inflammatory cytokines and nuclear factor (NF)-κB [35]. A dysregulated hypothalamic-pituitary-adrenal (HPA) axis could contribute to visceral hypersensitivity [36], which is typically seen in patients with IBS. Abnormal 5-HT functioning is associated with altered gut motility and enhanced nociceptive pain sensitivity [37], both symptoms characteristic of IBS. Interestingly, laboratory studies with chronically stressed mice have found that curcumin administration was able to reverse the effects of chronic stress on behaviour, the HPA axis and BDNF protein levels [38]. 

Another factor that could contribute to the therapeutic effect of curcumin is related to its poor oral bioavailability [20]. The majority of the curcumin ingested is excreted in the faeces unmetabolized [21]. This means that after ingestion, curcumin reaches the gut almost unaltered and could exert potentially beneficial effects on the gut and gut microbiota. In Sprague Dawley rats with hepatic steatosis (induced by a high-fat diet), curcumin not only restored intestinal barrier integrity (increased expression of tight junction proteins ZO-1 and occluding), it markedly altered the overall composition of the gut microbiota, towards that of lean rats maintained on a normal diet [39]. Also underlying the disease process of IBS is altered intestinal permeability, specifically, decreased expression of the tight junction proteins ZO-1 and α-cathenin [40]. Curcumin has demonstrated positive effects on intestinal permeability in animal models, improving the structure of intestinal tight junctions and upregulating the expression of occludin in the intestinal mucosa [41]. These could explain its positive effects on IBS symptomatology.

With regard to the possible side effects of curcumin use, human trials assessing its safety and toxicity have found it to be safe, with occasional reports of slight giddiness, nausea, and diarrhoea [42]. Encouragingly, no adverse events were also reported in any of the trials reviewed in this study [24,25,26,28]. However, at higher doses, curcumin may interact with some medications, such as anticoagulants [43]. Quality longitudinal studies are needed to confirm the safety of curcumin use. 

Finally, the limitations of our current study should be discussed. As only three studies were available for meta-analysis, a funnel plot or sensitivity analysis was not feasible. Second, inter-study variability in terms of curcumin formulation (even combined with peppermint oil, a known antispasmodic [44], in one study [26]) and the diagnosis of IBS likely contributed to the overall heterogeneity of the meta-analysis, and could have affected the reliability of current findings. Furthermore, curcumin was mixed with at least one other therapeutic or potentially therapeutic material in two of the trials analysed [26,28], hence, it is difficult to quantify a curcumin-attributable SMD and determine whether the therapeutic benefits observed in these two studies were due to curcumin. More robust clinical trials involving a standardized curcumin preparation and larger sample sizes are warranted. Finally, as IBS is often a chronic, relapsing condition [45], follow-up studies over a longer duration should be conducted to ensure that the clinical improvement observed with curcumin use would be stable in the long-term. Current studies ranged from only 8 to 18 weeks [24,25,26,28].

## 5. Conclusions

Current evidence suggests that curcumin has a positive albeit not statistically significant effect (compared to placebo) on IBS symptoms, alleviating pain and improving quality-of-life scores in patients with at least moderate symptom severity. With its unique anti-oxidant and anti-inflammatory activities and ability to modulate gut microbiota, it is a potentially useful addition to our armamentarium of agents for managing IBS. Curcumin also appears to be safe and well-tolerated, with no serious adverse events reported in any of the trials reviewed. However, current findings are based on a considerably limited evidence base with marked heterogeneity. More robust clinical trials involving a standardized curcumin preparation and larger sample sizes should be encouraged.

## Figures and Tables

**Table 1:** Studies included in the systematic review (arranged alphabetically by first author’s last name).

Author, Year	Study Design	Country	Sample Size	Curcumin Dose and Formulation	Study Duration	Diagnosis of IBS	Conclusions
Alt, 2017 [26]	Double-blind, placebo-controlled randomized trial	Germany	99	- IQP-CL-101 softgel (contains 330 mg proprietary blend of curcuminoids and essential oils, 70 mg fish oil, 15 mg peppermint oil and 8 mg caraway oil as well as 263 μg thiamine, 39 μg folic acid and 625 μg vitamin D3)	8 weeks	Rome III	Significant improvement in IBS symptoms, compared to placebo (*p* < 0.001). Improvements seen as early as 4 weeks into treatment. No serious adverse events reported.
Bundy, 2004 [24]	Partially blinded, randomized, two-dose trial	United Kingdom	207	- 72 mg (1 tablet) of a standardized turmeric extract daily (Cynara™ Turmeric, Lichtwer Pharma (UK) Ltd., Marlow, UK) - 144 mg (2 tablets) of curcumin	8 weeks	Rome II	Significantly reduced IBS symptomatology in both treatment groups after 8 weeks (*p* < 0.001). No serious adverse events reported.
Brinkhaus, 2005 [25]	Double-blind, placebo-controlled, randomized trial	Germany	106	- *Curcuma xanthorriza* 60 mg daily - *Fumaria officinalis* 1500 mg daily	18 weeks	Extensive clinical examination ruling out organic causes	Both herb-based monotherapy did not significantly improve IBS symptoms compared to placebo.
Lauche, 2016 [27]	Double-blind, placebo-controlled, randomized crossover trial	Germany	32	- 5 g of Ayurvedic powder mixture (curry (*Murraya koenigii*)), pomegranate (*Punica granatum*) and turmeric (*Curcuma longa* rhizome pulvis) in a 6:3:1 ratio) dissolved in 100 mL of warm water	4 weeks	Rome III	No significant difference between Ayurvedic preparation and placebo for IBS symptom severity (*p* = 0.26).
Portincasa, 2016 [28]	Double-blind, placebo-controlled, randomized trial	Italy	121	- Two capsules of CU-FEO (*Curcuma longa* 42 mg and *Foeniculum vulgare* 17.5 mg)	30 days	Rome III	Significant improvement in IBS symptoms (*p* < 0.001) and quality of life. No serious adverse events reported.

**Table 2:** Results of Cochrane collaboration’s tool for assessing risk of bias.

Study (Author, Year)	Sequence Generation	Allocation Concealment	Blinding	Incomplete Outcome Data	Selective Outcome Reporting	Other Bias
Alt, 2017 [26]	+	+	+	+	?	?
Bundy, 2004 [24]	+	?	-	+	?	-
Brinkhaus, 2005 [25]	?	+	+	+	?	?
Lauche, 2016 [27]	+	-	-	+	?	?
Portincasa, 2016 [28]	-	+	+	+	?	?

Key: + low risk of bias; - high risk of bias; ? unclear risk of bias.
